# Supplementary material for: Fecal Microbiota Transplantation in Irritable Bowel Syndrome: A Systematic Review and Meta-Analysis of Randomized Controlled Trials
Source: Int J Mol Sci. 2023 Sep 26;24(19):14562. doi: 10.3390/ijms241914562 (PMC10573019; doi:10.3390/ijms241914562)
Supplement: Supplementary file 1 [file ijms-24-14562-s001.zip › ijms-2551360-supplementary.pdf]

This is an additional file of the manuscript entitled **"Fecal microbiota transplantation in irritable bowel syndrome: A systematic review and Meta-analysis of Randomized Controlled Trials."**

PICO of the study

Participants	Adult patients with moderate-severe IBS according to Rome III criteria who received FMT
Experimental intervention	Fecal Microbiota Transplantation
Comparator	Adult patients with moderate-severe IBS according to Rome III criteria which did not receive FMT
Outcomes	Patients reported symptoms

Search Strategy

<u>Pubmed/Medline:</u>

((((((Irritable Bowel Syndrome[MeSH Terms]) OR (Irritable Bowel Syndrome[Title/Abstract])) OR (Irritable Colon[Title/Abstract])) OR (Mucous Colitis[Title/Abstract])) OR (Mucous Colitides[Title/Abstract])) OR (IBS[Title/Abstract])) AND ((FMT[Title/Abstract]) OR ((((((((((((((((((((((((((Fecal Microbiota Transplantation[MeSH Terms]) OR (Fecal Microbiota Transplantation[Title/Abstract])) OR (Fecal Microbiota Transplantations[Title/Abstract])) OR (Fecal Microbiota Transplant[Title/Abstract])) OR (Fecal Microbiome Transplantation[Title/Abstract])) OR (Fecal Microbiome Transplantations[Title/Abstract])) OR (Fecal Transplant[Title/Abstract])) OR (Fecal Transplants[Title/Abstract])) OR (Donor Feces Infusion[Title/Abstract])) OR (Donor Feces Infusions[Title/Abstract])) OR (Intestinal Microbiome Transplant[Title/Abstract])) OR (Intestinal Microbiome Transplants[Title/Abstract])) OR (Intestinal Microbiota Transfer[Title/Abstract])) OR (Intestinal Microbiota Transfers[Title/Abstract])) OR (Intestinal Microbiota Transplantation[Title/Abstract])) OR (Intestinal Microbiota Transplantations[Title/Abstract])) OR (Intestinal Microbiome Transplantation[Title/Abstract])) OR (Intestinal Microbiome Transplantations[Title/Abstract])) OR (Intestinal Microbiota Transplant[Title/Abstract])) OR (Intestinal Microbiota Transplants[Title/Abstract])) OR (Intestinal Microbiome

Transfer[Title/Abstract])) OR (Intestinal Microbiome Transfers[Title/Abstract])) OR (Fecal Microbiota Transfer[Title/Abstract])) OR (Fecal Microbiota Transfers[Title/Abstract])) OR (Fecal Transplantation[Title/Abstract])) OR (Fecal Transplantations[Title/Abstract])))

Embase:

('fecal microbiota transplantation'/exp OR 'fecal microbiota transplantation':ab,ti OR 'fecal microbiota transplantations':ab,ti OR 'fecal microbiota transplant':ab,ti OR 'fecal microbiome transplantation':ab,ti OR 'fecal microbiome transplantations':ab,ti OR 'fecal transplant':ab,ti OR 'fecal transplants':ab,ti OR 'donor feces infusion':ab,ti OR 'donor feces infusions':ab,ti OR 'intestinal microbiome transplant':ab,ti OR 'intestinal microbiome transplants':ab,ti OR 'intestinal microbiota transfer':ab,ti OR 'intestinal microbiota transfers':ab,ti OR 'intestinal microbiota transplantation':ab,ti OR 'intestinal microbiota transplantations':ab,ti OR 'intestinal microbiome transplantation':ab,ti OR 'intestinal microbiome transplantations':ab,ti OR 'intestinal microbiota transplant':ab,ti OR 'intestinal microbiota transplants':ab,ti OR 'intestinal microbiome transfer':ab,ti OR 'intestinal microbiome transfers':ab,ti OR 'fecal microbiota transfer':ab,ti OR 'fecal microbiota transfers':ab,ti OR 'fecal transplantation':ab,ti OR 'fecal transplantations':ab,ti OR fmt:ab,ti) AND ('irritable colon'/exp OR 'irritable colon':ab,ti OR 'irritable bowel syndrome':ab,ti OR 'irritable bowel syndromes':ab,ti OR 'mucous colitis':ab,ti OR 'mucous colitides':ab,ti OR ibs:ab,ti)

Study name	Odds ratio	Lower limit	Upper limit	p-Value	Relative weight
Johnsen	2.053	0.785	5.369	0.143	39.53
Lahtinen	1.250	0.404	3.866	0.698	28.67
Holster	7.000	0.568	86.321	0.129	5.79
Holvoet	3.537	1.081	11.571	0.037	26.01
	2.202	1.203	4.031	0.010	

Statistics for each study

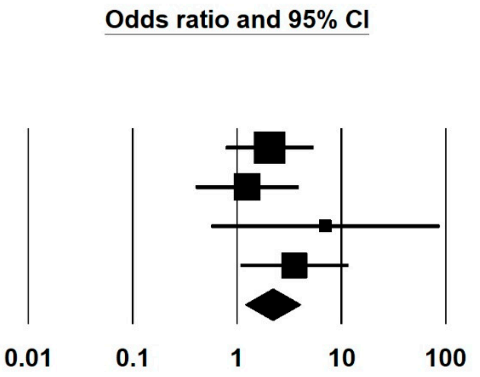

Figure S1. Forest plot of the subgroup analysis indicating the efficacy of the lower GI administered single dose FMT compared to autologous FMT as placebo.

Study name	Odds ratio	Lower limit	Upper limit	p-Value	Relative weight
Halkjær	0.150	0.040	0.559	0.005	37.65
Singh	0.190	0.025	1.432	0.107	15.96
Aroniadis	0.643	0.197	2.099	0.464	46.40
	0.306	0.137	0.686	0.004	

Statistics for each study

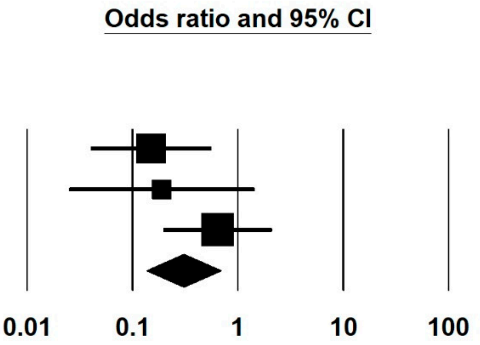

Figure S2. Forest plot of the subgroup analysis indicating the efficacy of the upper GI administered frozen FMT compared to non-FMT placebo.

Study name	Odds ratio	Lower limit	Upper limit	Z-Value	p-Value	Relative weight
Halkjær et al.	0.150	0.040	0.559	-2.827	0.005	47.99
Aroniadis et al.	0.643	0.197	2.099	-0.732	0.464	52.01
	0.320	0.077	1.328	-1.569	0.117	

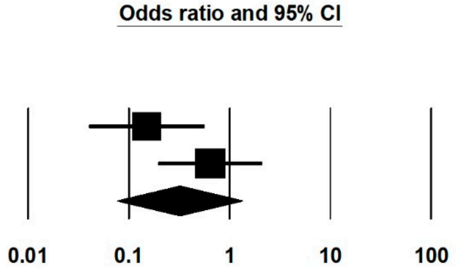

Figure S3. Forest plot of the subgroup analysis indicating the efficacy of the multiple doses administered FMT compared to non-FMT placebo.

Study name	Odds ratio	Lower limit	Upper limit	Z-Value	p-Value	Relative weight
Holster et al.	7.000	0.568	86.321	1.518	0.129	16.80
Lahtinen et al.	1.250	0.404	3.866	0.387	0.698	83.20
	1.670	0.596	4.676	0.976	0.329	

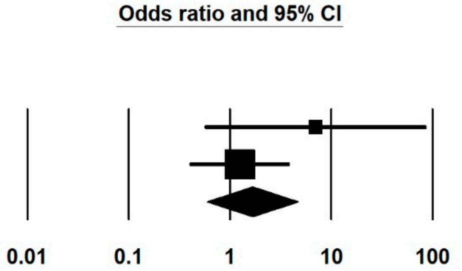

Figure S4. Forest plot of the subgroup analysis indicating the efficacy of the single donor FMT compared to autologous FMT as a placebo.

Study name	Odds ratio	Lower limit	Upper limit	Z-Value	p-Value	Relative weight
Johnsen et al.	2.053	0.785	5.369	1.466	0.143	60.31
Holvoet et al.	3.537	1.081	11.571	2.089	0.037	39.69
	2.547	1.207	5.375	2.454	0.014	

Statistics for each study — Odds ratio and 95% CI

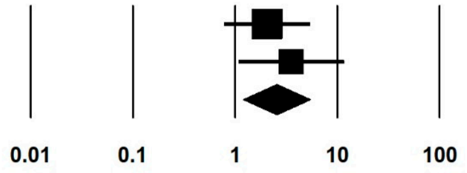

Figure S5. Forest plot of the subgroup analysis indicating the efficacy of the multiple-donor FMT compared to autologous FMT as a placebo.

Study name	Odds ratio	Lower limit	Upper limit	Z-Value	p-Value	Relative weight
Halkjær et al.	0.150	0.040	0.559	-2.827	0.005	70.23
Singh et al.	0.190	0.025	1.432	-1.611	0.107	29.77
	0.161	0.054	0.485	-3.248	0.001	

Statistics for each study — Odds ratio and 95% CI

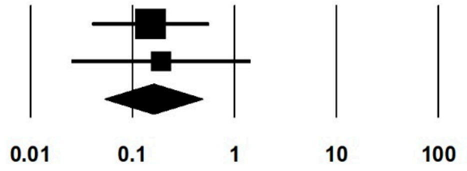

Figure S6. Forest plot of the subgroup analysis indicating the efficacy of the multiple-donor FMT compared to non-FMT placebo.
